# Using machine learning-based systems to help predict disengagement from the legal proceedings by women victims of intimate partner violence in Spain

**DOI:** 10.1371/journal.pone.0276032

**Published:** 2023-06-07

**Authors:** Elena Escobar-Linero, María García-Jiménez, María Eva Trigo-Sánchez, María Jesús Cala-Carrillo, José Luis Sevillano, Manuel Domínguez-Morales

**Affiliations:** 1 Architecture and Computer Technology department (ATC), Robotics and Technology of Computers Lab (RTC), E.T.S. Ingeniería Informática, Avda. Reina Mercedes s/n, Universidad de Sevilla, Seville, Spain; 2 Department of Experimental Psychology, Facultad de Psicología, C/Camilo José Cela s/n, Universidad de Sevilla, Seville, Spain; Guru Ghasidas Vishwavidyalaya: Guru Ghasidas University, INDIA

## Abstract

Intimate partner violence against women (IPVW) is a pressing social issue which poses a challenge in terms of prevention, legal action, and reporting the abuse once it has occurred. However, a significant number of female victims who file a complaint against their abuser and initiate legal proceedings, subsequently, withdraw charges for different reasons. Research in this field has been focusing on identifying the factors underlying women victims’ decision to disengage from the legal process to enable intervention before this occurs. Previous studies have applied statistical models to use input variables and make a prediction of withdrawal. However, none have used machine learning models to predict disengagement from legal proceedings in IPVW cases. This could represent a more accurate way of detecting these events. This study applied machine learning (ML) techniques to predict the decision of IPVW victims to withdraw from prosecution. Three different ML algorithms were optimized and tested with the original dataset to assess the performance of ML models against non-linear input data. Once the best models had been obtained, explainable artificial intelligence (xAI) techniques were applied to search for the most informative input features and reduce the original dataset to the most important variables. Finally, these results were compared to those obtained in the previous work that used statistical techniques, and the set of most informative parameters was combined with the variables of the previous study, showing that ML-based models had a better predictive accuracy in all cases and that by adding one new variable to the previous work’s predictive model, the accuracy to detect withdrawal improved by 7.5%.

## 1 Introduction

Violence suffered by women in intimate partner relationships (IPVW) is an important social problem that requires continuous and coordinated intervention from many different spheres. When prevention has not been possible and the violence has already occurred, the act of filing a complaint, regardless of who the complainant is, may trigger legal proceedings for IPVW, even when the victim refuses to participate in such proceedings (see “clarifications”). In Spain, only 21.7% of cases in which women have suffered violence from a current male partner have been reported to the police or at Court. The woman herself fileds the complaint in 80% of these cases, remaining 20% of complains were filed by another person aware of the offense [1]. According to official data in Spain, withdrawals from legal proceedings in IPVW cases occur in 9.86% of complaints. This refers to women who disengaged from prosecution making use of Article 416 of the Criminal Procedure Code [2], and 27.9% of women who withdraw at any other moment of the legal proceedings [1].

According to the literature on this topic, refusing to continue participation in the legal proceedings may have important consequences, such as the case not being prosecuted [3]. It can have a negative effect on general opinion, including professionals attending these women, increasing the sense of impunity of the accused, but also affecting the credibility of the victims [4]. At the same time, the professionals attending these women may feel that their work has been in vain [5], and the judicial system itself is affected by the increase in its costs. These consequences can increase victim-blaming attitudes and reinforce stereotypical beliefs about victims of IPVW. However, evidence has shown that withdrawing from prosecution in IPVW cases may stem from psychosocial processes related to the victim’s liberation and recovery from the violent relationship. Forcing the victim to participate with prosecution might have worse repercussions [6]. Results have underlined the importance of understanding women’s decision when they are involved with the Criminal Justice System for IPVW. Being to predict withdrawals is useful in these cases, not necessarily to prevent this from happening, but to advise, support, and refer women to the appropriate resources that help victims in their recovery process [6, 7]. When withdrawal is unavoidable or is considered the best option for a woman, understanding this phenomenon and the factors that predict it will helps reduce victim-blaming attitudes and potential secondary victimization. This requires comprehension and respect of the victims’ decision not to participate with prosecution [6]. In this sense, although respecting women’s agency is essential, preventing IPVW victims’ disengagement from prosecution is particularly important when it is a consequence of an inadequate response given by the legal system that may generate distrust in victims, or because of a lack of resources to face the legal proceedings.

It is therefore vitally important to be able to predict this disengagement, especially if it occurs out of fear or distrust of the justice system, in order to be able to intervene before it happens, advising and supporting women and providing them with the appropriate resources [7].

There have been several studies relating withdrawal from prosecution to different variables (see [5]). Some of the proposed predictive models have used secondary data, mainly drawn from cases dealt with in Domestic Violence Courts (e.g. [3, 8, 9]) and are sometimes limited to cases where only physical violence occurs [10]. More recently, some studies carried out in Spain have overcome these limitations by using data collected directly from the victims (e.g. [7, 11, 12]). These studies aimed to obtain, through the binary logistic regression (BLR) statistical technique, sufficiently complete and parsimonious models to predict disengagement from the legal proceedings, depending on the type of variables used and whether retrospective or prospective data were used. The last of the models developed, as discussed in [13], achieves a correct classification of 83.6% of the cases of disengagements with only six predictors: the victim perceiving that she is not making decisions jointly with her lawyer during the judicial procedure; the woman having thought about the possibility of returning to the abuser after filing the complaint; the requested protection order being denied; having contact with the abuser; feeling guilty after filing the complaint; and not receiving psychological support.

With the type of data used in these studies, a low number of experimental subjects, many variables with complex relationships between them, and/or no sufficient a priori knowledge of the relationships between these variables, it is known that Machine Learning (ML) methods could represent a better alternative to statistical techniques such as logistic regression [14–16]. In such cases, an approach based on training classifiers based on a subset of the available data, and then testing this classification using the rest of the dataset, may make better predictions than a classic statistical approach. This is the general aim of this paper: using different ML techniques to study to what extent they can predict female victims’ disengagement from the legal proceedings, with two specific objectives: a) comparing the predictive power of different ML techniques and with the previously obtained BLR models, in terms of general precision, sensitivity, and specificity; and b) comparing the relevant variables detected by the two approaches, ML and BLR, with the possibility of identifying new variables that could be useful regarding disengagement using ML methods.

## 2 Background

From a methodological point of view, some studies have already shown the usefulness of using alternatives to regression models (such as neural networks, support vector machines (SVM), or decision trees) given the difficulty, in practice, of complying with the statistical assumptions required for adequate development and interpretation of the regressions. Likewise, the use of artificial intelligence and, specifically, ML and the procedures indicated above, has interesting advantages over regressions. One of them is the possibility of working even in the presence of multicollinearity among the predictors, or of working with a large group of independent variables or “inputs”, in line with the approach of [17]. This work states that, in the presence of predictors with little predictive power, which are normally eliminated from the models, the accuracy of the prediction can be significantly increased thanks to the aggregation of variables performed by ML. Also, ML techniques could also be more appropriate when it is not possible to assume the existence of multicollinearity between variables [18].

Furthermore, in Social Sciences it seems that the use of ML may be advantageous over regressions in situations in which there are powerful predictors and little “noise” in the data analyzed; even in the opposite case the use of regression is at least as useful as ML both in the construction of the model and in its replication with empirical data [19]. The use of ML brings, in addition, the possibility of model testing by cross validation [20], something that to date has not been done with the regression models proposed by the authors of the papers cited above, whose purpose was to predict withdrawals using regression models. Even though cross validation is possible when using regression models, ML methods ease the quantification of uncertainty as well as making accurate predictions especially with small datasets [20].

Given these benefits, ML procedures have been gaining visibility and strength in Applied Social and Health Sciences. Some works have focused on predicting different types of crimes, and even the location of where they occur, using neural networks and Multilayer Perceptron (MLP) [21]. But there has also been a proliferation of works that aim to predict the type of IPV with Random Forest and SVM classifiers [22] or use neural networks to determine accurate predictors of this type of violence [23]. Furthermore, some ML techniques have been tested to assess the improvement in the accuracy of detection of false positives and false negatives in the assessment of risk for victims of IPVW with respect to protocols specifically designed for this purpose [18].

Regarding the decisions made in judicial proceedings, there are also works focused on the use of ML to predict judicial decisions in a wide variety of crimes, such as human rights violations, family and/or civil law cases, and even labor litigation, among others. Rosili’s review [24] lists these and other works that use different Machine Learning techniques to predict judicial decisions for different types of crimes. However, a recent exploratory literature search (see Table 1) has not identified any ML work targeting judicial decisions in the area of IPVW, nor studies using AI for predicting the decisions of victims of this type of violence, including the specific decision to continue or not in a judicial proceeding against the aggressor. Therefore, to the authors’ knowledge, this is the first work using artificial intelligence techniques to predict the decision of victims of IPVW not to continue with a legal proceeding initiated against the aggressor.

**Table 1:** Exploratory literature search on machine learning to predict withdrawal from prosecution in IPVW cases.

Database	Web of Science
Date of search	April 8th 2022
Search strategy*	TS=(“intimate partner violence” OR “domestic violence” OR “dating violence” OR “violence against wom*n”) AND TS=(predict* OR forecast*) AND TS=(Withdrawal OR cooperation OR “drop* charge*” OR disengage*) AND TS=(“legal proceedings” OR “criminal justice system” OR “legal cooperation” OR “legal system”) AND TS=(“machine learning” OR “artificial intelligence”)
Year of publication**	Any study before 2022

*Notes*:

*This search found no results;

**No other filters were used on Web of Science

## 3 Materials and methods

In this section, the components and techniques followed through the development of the study are presented. In the first place, the dataset used is exposed in detail, explaining the data collection process and the summary of participants and variables obtained. In the next sub-section, the complete data pre-processing and processing is presented.

As an introduction, this process involves a first phase of data cleaning, data augmentation and data wrangling, which consist of removing erroneous data, replacing missing values by correct ones following specific techniques, and changing raw data into a more valid format, respectively. In the second phase, a grid search process is carried out for different classifiers, so that the optimization of their hyperparameters can be obtained, and the classification between disengagement and non-disengagement can be more accurate.

The following phase is the study of the most useful variables for each classifier, which is based on understanding the process performed by the AI classifiers to obtain the classification results (as most of them are like ‘black boxes’ and the decision process is not clear). To do so, recent works use different techniques that are part of the field known as “Explainable Artificial Intelligence” (xAI) or “Explainable Deep Learning” (xDL). A summary of the most recent techniques can be found in [25].

**Fig 1:**
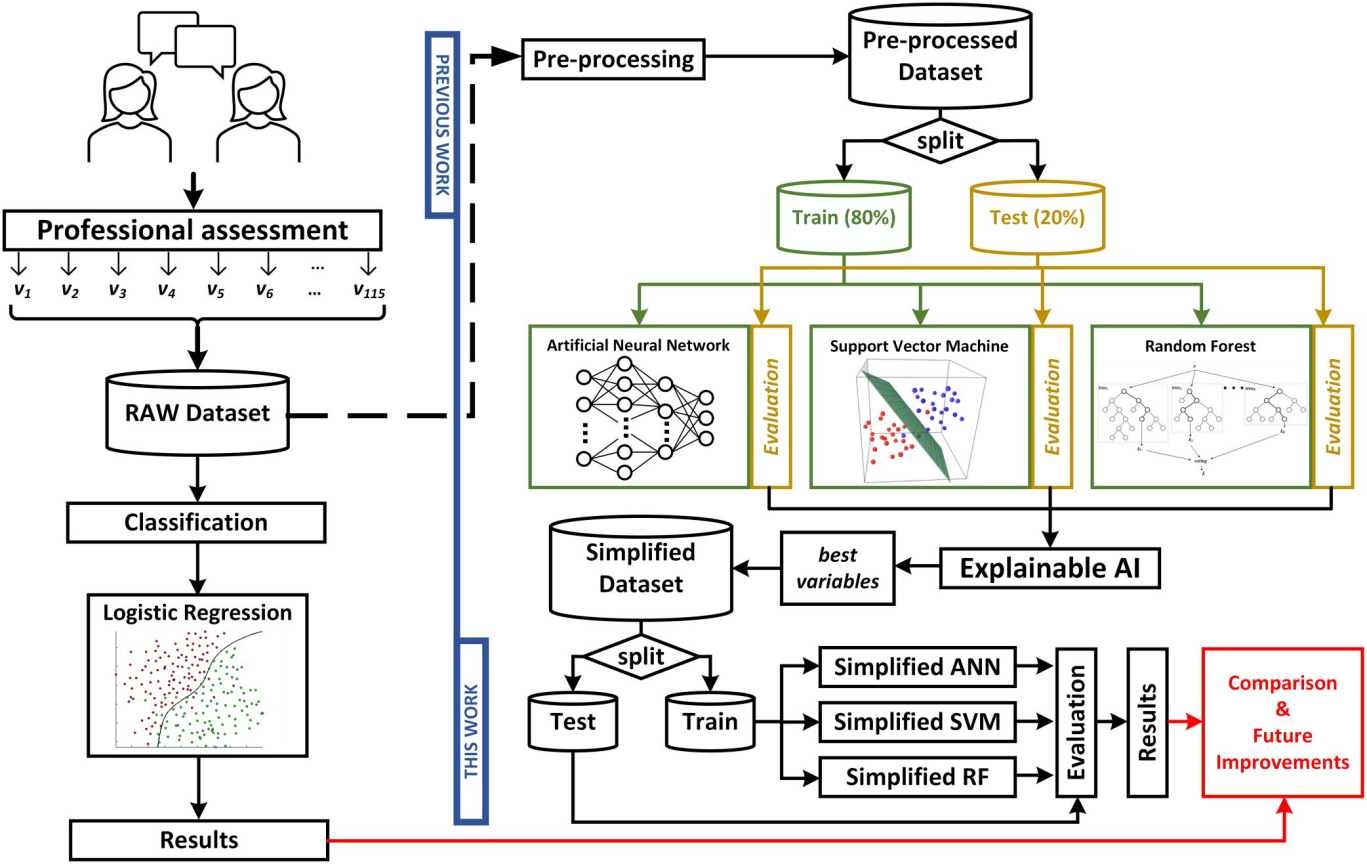
Graphical abstract. Left side corresponds to the previous work, whose results are compared with the ones obtained in this work (right side).

Finally, the evaluation metrics for each model are obtained to perform a suitable assessment. The graphical abstract of this work can be observed in Fig 1.

### 3.1 Dataset

The dataset was obtained from a survey conducted in previous studies (for a more detailed description of the sample and the procedure of data collection [7, 11, 12]). The total sample was 763 women victims of IPVW that participated in the legal proceedings against their partners or ex-partners in southern Spain (this dataset can be publicly accessed through https://github.com/mjdominguez/LPIPV). They were interviewed in the Andalusian Victims Assistance Service (SAVA in its Spanish acronym), Sheltered Housing, Municipal Centers for Information for Women (CMIM), and some victims help foundations. The average age of the participants was 35.60 (*SD* = 11.09). Out of the total sample, the variable of interest (disengaging or not from the legal proceedings) was first known for 49.5% of the women whose legal cases had been finalized when the data collection started (retrospective data). For the remaining 50.5% of the cases, this variable was not known until the legal proceedings had ended (prospective data). Overall, 32.2% of women did disengage from the legal proceedings, a higher percentage than in the population because of an intentional attempt to balance the group of participants in both groups.

There were 116 variables included in the dataset (115 after detaching the output variable, named in AI systems as label) before data pre-processing and the posterior creation of dummy variables for analytic purposes (all the variables are detailed in S1 Appendix). They were extracted from the scientific literature on the topic and interviews with victims and professionals, until information saturation about possible motives for disengaging was reached. These variables are grouped into three sets. The first and second sets refer to sociodemographic (e.g., age or number of children) and psychological, emotional and motivational variables (e.g., feelings of guilt after filing the complaint or receiving psychological support), respectively. Both groups were extensively described in [7, 11]. The third set (see [12]) was related to variables referring to the legal proceedings and professionals involved (e.g., applying for a protection order, whether the protection order was denied).

### 3.2 Pre-processing phase

As mentioned in the introduction of this section, the first phase of the data pre-processing was the data cleaning, data augmentation and data wrangling. This is an essential step for AI classification techniques, since automatic classifiers cannot have any missing values in their input data, as well as the data format has to be appropriate and easy to process to reach a more effective classification. To this end, it is necessary to analyze each sample and apply specific techniques of data pre-processing.

Firstly, the entire dataset was analyzed, and we found that 32 samples had missing values for the disengagement variable. In this case, as this variable is the actual label for prediction and a classifier cannot be trained without labels, the samples had to be removed from the dataset.

Next, we observed that each of the 731 resulting samples (763 initials—32 with missing label) had a missing value in some variables. Moreover, of the 115 initial variables (the 116th variable was the label), only 20 of them had all the samples, with approximately 80% of variables having a missing value. In view of this, missing values of samples or variables cannot simply be removed, since the final dataset would lose large amount of information and the pre-processing would not be valid in order to obtain a reliable classification. Thus, after removing the samples with missing labels, the variables with more than 15% of missing values were deleted, since it has been shown that higher percentages of missing values may impact severely on subsequent interpretation [26]. After this step, the 115 variables were reduced to 47. In order to make the following steps easier, those variables were categorized to numerical values.

At this stage, we entered in the phase of treating missing data. This can be done through data augmentation, with specific methods of value imputation. Specifically, in this study, the mean method was applied, in which missing values are replaced with the average of the variable values [27]. For this step to be more precise, the dataset was divided into two subsets: one with the samples with the disengagement label, and the other subset with samples of the non-disengagement label. Thus, the average of each variable was calculated according to the label value, preserving possible information and patterns of the data related to the labels.

The last step of the data pre-processing phase was data wrangling, in which each variable was converted to dummy variables. Dummy variables are the conversion of a categorical variable into a group of variables with values of 0 and 1. In this way, if a categorical variable takes three unique values, there will be three dummy variables to represent it, with values of 1 when the category is present in the corresponding dummy variable. This conversion facilitates the information patterns that the classifier will find in the training process, as well as providing a high flexibility to apply different AI models. In general, dummy variables enable an easy implementation, use and interpretation of the final application [28]. With this step, taking categorical variables to obtain their dummy variables, the final dataset had 93 variables. The summary of the final resulting dataset after data pre-processing is illustrated in Table 2.

**Table 2:** Summary of raw and processed dataset.

	Original	Final
Samples	763	731
Variables	115	93

### 3.3 Classifiers

In this work, several classifiers were designed and tested using artificial intelligence techniques to determine whether the decision about abandoning the judicial process in women victims of abuse can be anticipated based on the parameters collected in the dataset.

For this purpose, three known classifiers were used: Random Forest [29], Support Vector Machine [30] and Artificial Neural Network [31]. With all of them, a process of searching for the best possible combination of hyperparameters was carried out (named as “grid search”), performing hundreds of trainings for each classifier and searching for the best classification result on the test subset. The results concerning the best combinations will be shown in the following sections.

Once the best classifiers had been obtained, the most important part of this work was to determine which parameters (variables) in the dataset were the most important to determine the classification. To do this, with the trained classifiers, we extracted the percentages of participation of each parameter in the final results.

For the Random Forest, this can be determined directly by the characteristics of the classifier obtained, but for Support Vector Machine and Artificial Neural Network, this process requires the application of Explainable Deep Learning techniques to elucidate the participation of each parameter in the final result. The xDL technique used is based on the application of the “Anchors” model [32] as a model distillation technique on the dataset. In this technique, commonly applied on images, parts of the image are sequentially removed and their classification behavior is observed.

In our case, the same mechanism will be used but applied to the parameters of the dataset: the parameter values will be modified one by one by a negative value (unrelated to the information contained) and the changes in the various metrics obtained after classification will be observed. In this way, the parameters that have the greatest impact will be obtained based on those that cause the greatest decrease in the metrics evaluated.

Once the best parameters of each model are known, training will be carried out with various simplified models and combinations between them (including as input only the parameters previously selected). The results obtained after this analysis will allow us to know definitively the importance of these parameters in the final classification.

As a last aspect, and based on the results of a previous study carried out with statistical techniques [7], the parameters selected from previous studies will be used with the models developed in this work and combined with the best parameters of this study, in order to extract those parameters that were not taken into account in the previous study, but that could improve the final classification (in order to be used in subsequent studies).

The full process is as follows:

- Phase 1: Obtaining the best classification results using a grid search for each classifier.
- Phase 2: Identifying the best parameters for each classifier, applying explainable AI techniques.
- Phase 3: Simplifying the classifiers using only the best parameters as input.
- Phase 4: Using the parameters obtained in the previous works to compare the results.
- Phase 5: Combining the parameters selected in the previous work with the best parameters of this work.

In the next section, we will begin by describing the various classifiers designed for this work and, subsequently, the techniques and metrics used to evaluate them.

#### 3.3.1 Artificial neural network

The first classifier is an Artificial Neural Network, more precisely a multilayer perceptron (MLP). MLP models have an input layer with a number of neurons equal to the number of variables of the dataset, an output layer with a number of neurons equal to the classes to classify and, between these layers, an unspecified number of hidden neurons and layers. Moreover, in this MLP architecture, the neurons of a layer are fully connected to the next layer.

The number of hidden layers and neurons is a hyperparameter that has to be chosen. To choose the optimal number, a comprehensive search between a different number of neurons and hidden layers is carried out. In turn, to obtain an accurate prediction, the MLP implements an activation function in each neuron considering the value of the previous connected neuron and a random weight that changes in order to obtain a minimal error in the output neurons. For this computation, several parameters can influence the final value of the actual neuron and with it the final prediction. To obtain an optimal value of the hyperparameters, a grid search process has to be followed, combining different hyperparameters to obtain an optimal model configuration. Firstly, the learning rate is the hyperparameter that determines the update rate of weights in each neuron, known as the step size, and usually takes values between 0 and 1. A smaller learning rate indicates very slow changes on the weights, while higher values indicate the opposite. In the grid search process, learning rate values between 1e-2 and 1e-5 are analyzed. On the other hand, dropout rate is the percentage of neurons randomly ignored by the ML algorithm in the training phase. This is done to prevent the overfitting of the classifier, which is the effect of knowing to well the prior data, with the result that it affects negatively on the predictions over new data. In this way, higher values of dropout rate can result in a more generalized model, although values that are too high can lead to an underfitted model. In the hyperparameter optimization process, the dropout rate varies between 0.1 and 0.3. Once these parameter values that influence the result of each node are chosen, the ML model can be trained over the dataset. However, it is essential to choose between different batch sizes to obtain a better prediction accuracy. Batch size is the parameter that defines the number of samples that will be introduced in the model before updating any other hyperparameter. Higher batch sizes indicate fewer changes in the parameters, while with lower sizes more updates are performed, but with the risk of overfitting the model. To choose correctly the batch size of the training step, values between 4 and 32 are analyzed for different parameter combinations.

Overall, different numbers of hidden layers and neurons with different values of learning rate, dropout rate and batch size are combined in the grid search process to obtain an optimized configuration of all parameters. A total of 1248 combinations are analyzed. Table 3 illustrates the possible values for every hyperparameter.

**Table 3:** Grid search process for ANN classifier.

**Neurons and layers**	[2, 3, 4, 5, 6, 7, 8, 9, 10, 11, 12, 13, 2:4, 3:6]
**Learning Rate**	[1e-2, 1e-3, 1e-4, 1e-5]
**Dropout Rate**	[0.1, 0.2, 0.3]
**Batch Size**	[4, 8, 12, 16, 20, 24, 28, 32]

#### 3.3.2 Support Vector Machine

The second classifier is a Support Vector Machine (SVM), which is an algorithm that searches for a hyperplane in a space of dimension of the number of input variables to separate the data in the different classes. In addition to the hyperplane, it is common to choose the maximal margin hyperplane, which consists of using the closest data points to the hyperplane, known as support vectors, to maximize the distance between those support vectors and the hyperplane [33]. In this way, classification of new data can be more accurate. Another aspect to take into account is that the data is not usually linearly separable, making it difficult to establish a hyperplane correctly separating every data point without having an overfitted model. To overcome this, the model has to add a soft margin that allows the trained model to misclassify some data points. Both margin distance and misclassified points are controlled by C parameter or Regularization parameter. For high values of C parameter, more penalty is applied to each misclassified point, allowing very few misclassifications, and a small distance between the hyperplane and support vectors is chosen to avoid misclassifications. The opposite occurs for smaller values of C parameter. In the grid search process for the optimization of the SVM configuration, C parameter values from 0.01 to 1000 are analyzed.

On the other hand, in SVM the non-linearly separable data can be treated with a transformation using kernel functions. An appropriate kernel function maps the original data dimension into a higher dimensional space, known as feature space, so that the data points can be separated more easily. In more detail, the kernel function uses as input the original features and obtains a similarity measure in the new space as output, where similarity refers to the closeness degree of each data point [34].

In this transformation and to control the distance between one point and its neighbor, the gamma parameter is used. Higher values of gamma indicate that the closeness of the next point is very small so that it can be included in the same class. In the optimization process, gamma parameter values between 1*e* − 4 and 1 are analyzed. Moreover, in this grid search phase, three kernel functions are used: Radial Basis Function kernel (RBF), polynomial kernel and sigmoid kernel.

In short, different values of C parameter, gamma parameter and different kernel functions are combined in 90 possibilities to obtain an SVM classifier with an optimized configuration. Table 4 shows all values used for each hyperparameter.

**Table 4:** Grid search process for SVM classifier.

**C**	[0.01, 0.1, 1, 10, 100, 1000]
**Gamma**	[1, 0.1, 0.01, 0.001, 0.0001]
**Kernel**	[RBF, Polynomial, Sigmoid]

#### 3.3.3 Random forest

The third and last classifier used is a Random Forest. This model consists of an ensemble of decision trees, where each tree obtains a random vector sampled independently and with the same distribution as every tree in the forest, having uncorrelated tree models [35]. Some settings are essential to obtain an accurate RF model. The first one is the number of estimators, referring to the number of decision trees in the forest. The more estimators the RF model has, the higher the accuracy that can be reached; however, a high number of decision trees can influence badly on the computing load. In this study, from 100 estimators to 1000 estimators with a step of 100 are applied to the RF model.

Another parameter to be considered is the maximum number of features that an individual tree can use. In this case, two size restrictions can be used: the first, where the tree automatically uses the features that naturally make sense, without having a maximum size; and the second, where the maximum size is the square root of the original features. Higher values of features can positively affect the accuracy, since each tree has more options to consider; however, it can negatively affect on the diversity of each tree.

On the other hand, the maximum depth of each decision tree has to be established, which indicates the splits that the tree has internally. A higher number of splits leads to more information being considered, but can also lead to overfitting issues. In the grid search process, the maximum depth analyzed is between 0 and 110 with steps of 10.

The minimum number of samples that can be used to allow the tree to split a node also needs to be considered. With a higher number of this parameter, the node has to consider more samples to make a decision and then pass to the next split. In this case, values evaluated in the grid search are from 2 samples to 10 out of the 731 total samples. Similarly, the number of minimum samples that needs to be on the leaf node has to be established. The leaf nodes are the terminal nodes of the tree, where the decision of belonging to a specific class is taken. In this case, the minimum samples between 1 and 4 are analyzed. In both parameters, reaching higher values lead to an underfitting problem, where the algorithm cannot identify patterns on the training data and cannot make any correct classification.

Finally, it is necessary to consider whether or not to apply the bootstrap aggregation or bagging, which is a method that randomly select samples and allow the replacement. Considering all the settings mentioned, the grid search can be carried out over more than 4000 combinations. However, to reduce the computational load, a randomized grid search is conducted, with 100 random combinations to find an optimal RF configuration. Table 5 illustrates all hyperparameter values.

**Table 5:** Grid search process for RF classifier.

**Estimators**	[100, 200, 300, 400, 500, 600, 700, 800, 900, 1000]
**Max. Features**	[Auto, Root square]
**Max. Depth**	[10, 20, 30, 40, 50, 60, 70, 80, 90, 100, 110, None]
**Min. Samples to Split**	[2, 5, 10]
**Min. Samples for Leaf**	[1, 2, 4]
**Bagging**	[True, False]

### 3.4 Evaluation procedure

To evaluate the effectiveness in the classification results of a classifier, the most common metrics are used: accuracy (most-used metric), sensitivity (known as recall in other works), specificity, precision, and F1_*score*_ [36]. To this end, the classification results obtained for each class are tagged as “True Positive” (TP), “True Negative” (TN), “False Positive” (FP) or “False Negative” (FN). According to them, the high-level metrics are presented in the next equations:

$$\begin{array}{c} Accuracy=\sum_{c}\frac{TP_{c}+TN_{c}}{TP_{c}+FP_{c}+TN_{c}+FN_{c}},c\in classes \end{array}$$ (1)

$$\begin{array}{c} Sensitivity=\sum_{c}\frac{TP_{c}}{TP_{c}+FN_{c}},c\in classes \end{array}$$ (2)

$$\begin{array}{c} Specificity=\sum_{c}\frac{TN_{c}}{TN_{c}+FP_{c}},c\in classes \end{array}$$ (3)

$$\begin{array}{c} Precision=\sum_{c}\frac{TP_{c}}{TP_{c}+FP_{c}},c\in classes \end{array}$$ (4)

$$\begin{array}{c} F1_{score}=2*\frac{precision*sensitivity}{precision+sensitivity}. \end{array}$$ (5)

About those metrics:

- Accuracy: all samples classified correctly compared to all samples (see Eq 1)
- Sensitivity (or recall): proportion of values classified as “true positive” that are correctly classified (see Eq 2)
- Specificity: proportion of values classified as “true negative” that are correctly classified (see Eq 3)
- Precision: proportion of values classified as “true positive” in all cases that have been classified as it (see Eq 4)
- F1_*score*_: It considers two of the main metrics (precision and sensitivity), calculating the harmonic mean of both parameters (see Eq 5)

The above metrics are common to all ML/DL systems. Therefore, the classifier systems developed in this work will be evaluated according to all the metrics detailed in this subsection. Moreover, the results obtained for the classification system, will be compared with the results obtained in previous works.

The results obtained for the previous metrics will be used not only for obtaining the best model for each classifier (phase 1), but to evaluate the classifiers during the xAI application, to compare the different classifiers with the previous work and to obtain the final results of the work (in short, in all of the phases described above).

## 4 Results and discussion

Following the process detailed phase-by-phase in the previous section, the best results for each classifier are presented below, followed by the application of xAI techniques to extract information from the classifiers and elaborate the final one based on the parameters that provide the most information to the system. Finally, these parameters are combined with those used in the previous work.

### 4.1 Best result for each classifier

After the grid search process, the best candidates can be extracted. Due to the nature of the social problem, the most important aspect was to correctly identify those women who were going to drop out of the judicial process, since these were the cases on which action could be taken to help them. Because of this, the parameter that would mark the goodness of the system would be the sensitivity of the disengagement class.

After the evaluation, for ANN and RF classifiers only one candidate was obtained as the optimized model; however, for the SVM classifier, three candidates were obtained with the same results but different parameter configurations.

The best candidate for the ANN classifier was formed by one hidden layer with 6 neurons, with a dropout rate of 0.1, a learning rate of 1e-3 and a batch size of 4 samples.

The best RF model consisted of 1000 estimators, with each of them having a maximum number of features equal to the square root of the original number of features. On the other hand, the minimum samples in each node to allow the split of the tree and the minimum samples in the final nodes were 5 and 2, respectively. Moreover, the bootstrap aggregation method was not applied in this optimized configuration.

Finally, for the SVM classifier, three possible optimal configurations were found, named SVM_1_, SVM_2_ and SVM_3_. All of them had in common the application of the RBF kernel, with the gamma parameter value of 0.1. However, SVM_1_ has a C parameter value of 100, SVM_2_ has 1000, and SVM_3_, 10. In Table 6, these optimal model configurations along with the evaluation results are illustrated.

**Table 6:** Best model results for each classifier.

	Hyperparameters	Accuracy	Sensitivity	Specificity	Precision	F1-score
**ANN**	6 neurons DP: 0.1 LR: 1e-3 BS: 4	91.84	0.7576	0.9649	0.8621	0.8065
**SVM_1_**	C: 100 Gamma: 0.1 Kernel: RBF	87.76	0.8182	0.8947	0.6923	0.75
**SVM_2_**	C: 1000 Gamma: 0.1 Kernel: RBF	87.76	0.8182	0.8947	0.6923	0.75
**SVM_3_**	C: 10 Gamma: 0.1 Kernel: RBF	87.76	0.8182	0.8947	0.6923	0.75
**RF**	Estimators: 1000 Depth: 60 Features: sqrt Split samples: 5 Leaf samples: 2 Bootstrap: no	88.44	0.7576	0.9211	0.7353	0.7463

The table shows that the best global accuracy value was obtained for the ANN model. Moreover, the specificity, precision and f1-score values for the ANN classifier was higher than all the other models, indicating that the model was better at correctly classifying non-disengagement labels, as well as being more consistent and with a better performance over the unbalanced data. However, the model reached a lower sensitivity than SVM models, which is an important metric, since it indicates how well the model classifies the samples as belonging to the disengagement class. For this study, one option was to have a model that correctly identified the cases in which the subject disengages from judicial process, rather than one which correctly classified samples as non-disengagement from the process. In this way, all three SVM models were better options for this prediction, even if the accuracy value obtained was lower than with the ANN and RF models.

**Fig 2:**
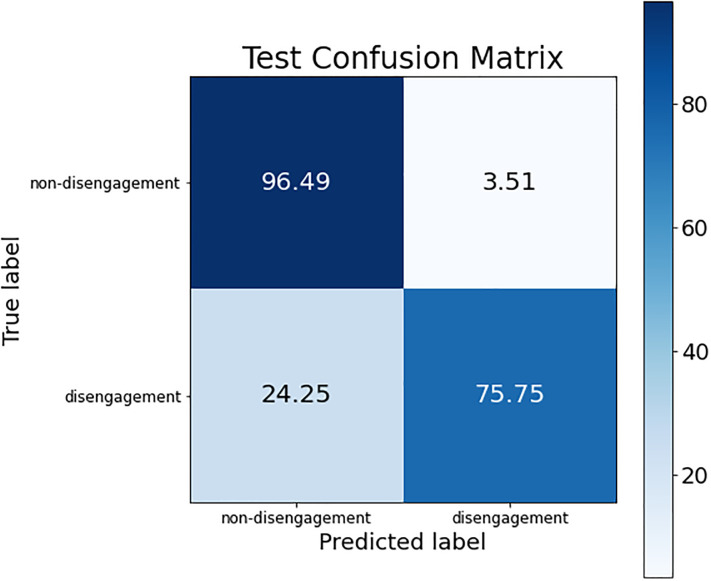
Confusion matrix for the best ANN model.

**Fig 3:**
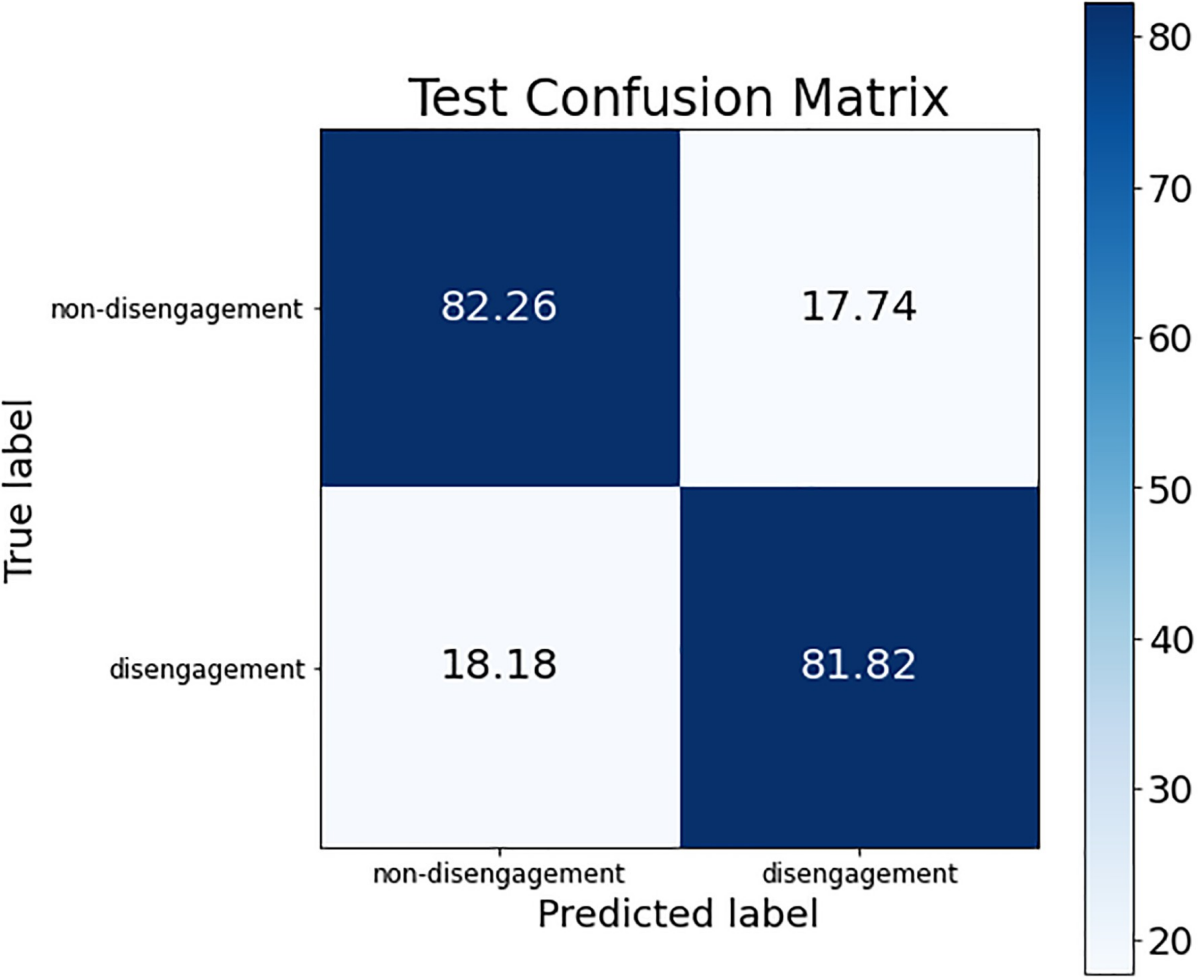
Confusion matrix for the best SVM model.

**Fig 4:**
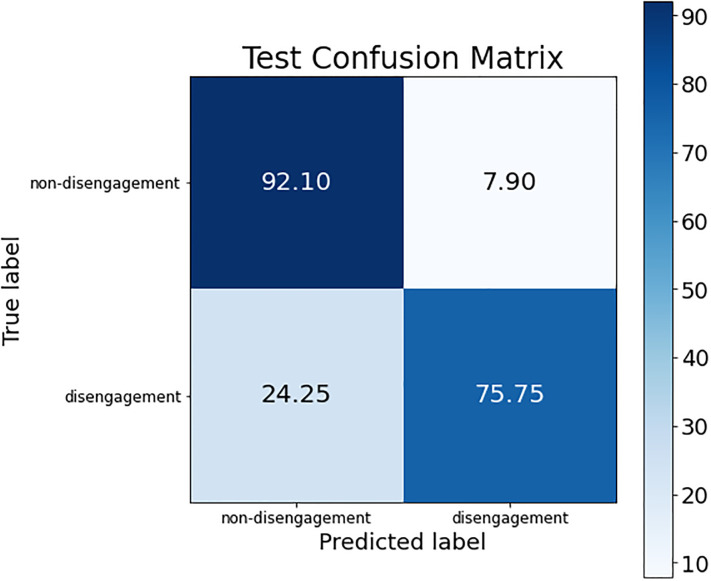
Confusion matrix for the best RF model.

These conclusions can be verified by looking at the confusion matrices of each of these models. The confusion matrix of the best ANN model is shown in Fig 2; the confusion matrix for the three SVM models (the same is obtained for the three of them) is presented in Fig 3; and, finally, Fig 4 shows the confusion matrix of the best RF model.

The difference regarding the sensitivity metric detailed before can be observed in the confusion matrices. The percentage of women who dropped out of the judicial process and are classified as such was higher in the SVM models than in ANN and RF (see the box at the bottom right of each confusion matrix), obtaining an improvement around 6% (although the non-disengagement class obtained a drop of accuracy around 14%).

### 4.2 xAI results

Once the best candidates had been obtained for each classifier, the most informative parameters were extracted for each model. This could be done through the application of the ‘Anchors’ technique (one of the multiple xAI techniques), as explained in the previous section.

The most important variables for the ANN classifier were computed as the difference of accuracy value when the variable was omitted. These results showed that 6.4% of the 93 original variables did not have any importance for the classifier (or had a negative influence on it), whereas the 93.6% remaining variables had minimal importance for the model. In more detail, the majority of variables had an individual influence of changing original accuracy by less than 5%, while 15% of variables influence in decreasing it by more than 5%. Specifically, 5 of them influenced in a decrease of 6.8% of the initial accuracy, and they were considered to be the most informative variables. These variables were: contact with the aggressor; having a protection order; current questionnaire; considering dropping out of the judicial process and the expectations that the abuser will be imprisoned. However, as explained previously, the variables were initially converted to dummy variables, having one variable of contact with the aggressor and another for not having contact with him. The same occurred for the protection order variable: one variable was obtained for having the order and a second one for not having a protection order. Considering these dummy variables, there were actually 7 variables that influenced in the highest decrease of the accuracy.

On the other hand, for the first SVM candidate, the feature importance process showed that 27 variables had a positive influence on the model; while 43 variables did not have any influence on the accuracy result, and the remaining 23 variables influenced negatively. Similarly, for both the second and third SVM models, 31 variables had a high importance, 39 did not have any importance, and 23 of them had a negative impact on the model.

Finally, for the RF model, results showed that 30 variables had a strong positive impact on the decision of the classifier, while 41 of them did not influence the model, and 23 of them were even less important, having a negative importance.

These results are summarized in Table 7.

**Table 7:** Variables filtering results obtained for all candidates, classified as positive influence (POS), neutral or minimal influence (NM) and negative influence (NEG).

	POS	NM	NEG
ANN	7	80	6
SVM_1_	27	43	23
SVM_2_	31	39	23
SVM_3_	31	39	23
RF	30	41	22

In the original study, the aim was to determine the minimum number of variables involved in the woman’s decision to abandon the judicial process. Sticking to this premise, we sought the model that required the fewest variables for this purpose. Due to this circumstance and observing the results in Table 7, the ANN-based model seemed to meet this requirement; however, it remains to be determined whether good results were obtained by eliminating all the variables that have not been identified as having a positive influence in each model. This process was carried out as follows.

### 4.3 Simplification of the classifiers

Considering only the most important variables for each classifier (tagged as ‘POS’ in Table 7), a new training phase was carried out by reducing the original dataset to one with the 7 most important variables for the ANN model in the first place. With this new dataset, new evaluation metrics were obtained for each of the five best models (see Table 8).

**Table 8:** Evaluation metrics for each classifier with set of ANN’s most important features.

	Accuracy	Sensitivity	Specificity	Precision	F1-score
**ANN**	90.48	0.8182	0.9298	0.7714	0.7941
**SVM_1_**	86.39	0.8788	0.8596	0.6444	0.7436
**SVM_2_**	87.07	0.7879	0.8947	0.6842	0.7324
**SVM_3_**	87.07	0.7879	0.8947	0.6842	0.7324
**RF**	87.76	0.7879	0.9035	0.7027	0.7429

Following the same steps, others datasets were obtained for the remaining sets of most informative parameters. Table 9 illustrates the results for the classification of each model over the 27 best SVM_1_ variables; Table 10 shows those evaluation metrics obtained with the dataset formed by the 31 most important features for SVM_2_ and SVM_3_ models; and Table 11 presents the results using the dataset composed of the best 30 features obtained with the RF classifier.

**Table 9:** Evaluation metrics for each classifier with set of SVM_1_’s most important features.

	Accuracy	Sensitivity	Specificity	Precision	F1-score
**ANN**	91.16	0.8485	0.9298	0.7778	0.8116
**SVM_1_**	89.12	0.8182	0.9123	0.7297	0.7714
**SVM_2_**	86.39	0.8485	0.8684	0.6512	0.7368
**SVM_3_**	87.07	0.8182	0.8860	0.6750	0.7397
**RF**	88.44	0.7576	0.9211	0.7353	0.7463

**Table 10:** Evaluation metrics for each classifier with set of SVM_2_ and SVM_3_’s most important features.

	Accuracy	Sensitivity	Specificity	Precision	F1-score
**ANN**	89.80	0.7879	0.9298	0.7647	0.7761
**SVM_1_**	89.12	0.8182	0.9123	0.7297	0.7714
**SVM_2_**	86.39	0.8182	0.8772	0.6585	0.7297
**SVM_3_**	82.99	0.7879	0.8421	0.5909	0.6753
**RF**	89.12	0.7273	0.9386	0.7742	0.75

**Table 11:** Evaluation metrics for each classifier with set of RF’s most important features.

	Accuracy	Sensitivity	Specificity	Precision	F1-score
**ANN**	89.80	0.7879	0.9298	0.7647	0.7761
**SVM_1_**	84.35	0.6667	0.8947	0.6471	0.6567
**SVM_2_**	82.99	0.6970	0.8684	0.6053	0.6479
**SVM_3_**	82.99	0.7273	0.8596	0.6	0.6575
**RF**	85.71	0.7273	0.8947	0.6667	0.6957

As can be seen in the previous tables, the best classification results were obtained by training the new reduced ANN model using as input only the most important variables of the previous SVM_1_ model (27 parameters), presented in the first line of Table 9.

The overall results showed that the resulting accuracy was practically identical to the best obtained in the initial models, but a very interesting phenomenon occurred: by reducing the parameters that influenced negatively or minimally the initial model, the sensitivity value for the new ANN model increases drastically and outperformed the initial SVM models. Thus, the metric used to measure the goodness-of-fit of the system improved by almost 10% with respect to the initial ANN model.

However, the number of entries was a negative aspect to take into account, as 27 parameters was a very large number for the initial social study. But, if we look at the results obtained by the new ANN model trained with the best parameters of the initial ANN model (only 7 parameters) shown in the first line of Table 8, the results did not differ much from those of the best case (a reduction of less than 0.7% in accuracy and 3% in sensitivity). Although this was an aspect to be taken into account, when looking for the simplest possible model, this model could be a strong candidate.

**Fig 5:**
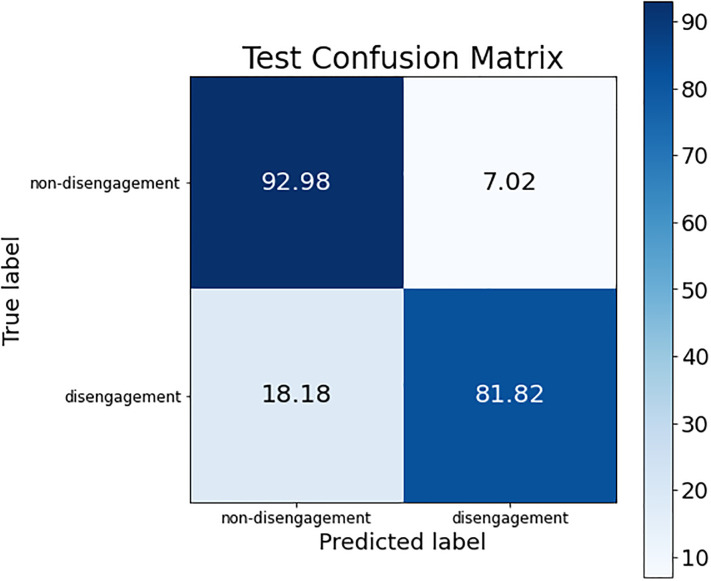
Confusion matrix for ANN model trained with the best 7 parameters from the initial the ANN model.

**Fig 6:**
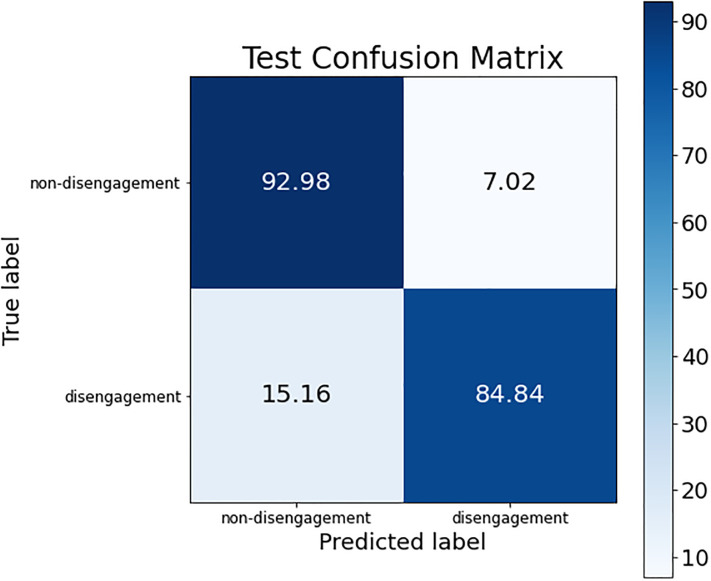
Confusion matrix for ANN model trained with the best 27 parameters from the initial the SVM_1_ model.

So, after this simplification process, the ANN model was the best candidate in both cases (with 7 and 27 parameters). Their confusion matrices can be seen in Fig 5 for the 7-parameter model, and in Fig 6 for the 27-parameter model.

If we look at the main differences between the two simplified models, it can be appreciated that the percentage of samples correctly classified for the non-disengagement class was exactly the same, but for the disengagement class there are differences: the 27-parameter model correctly classified 84.84% of the dataset’s samples for this class, while the 7-parameter model classified 81.82% (this represents a difference of 3% between them).

However, as the dataset used did not have a very large number of samples and the test subset represented 20% of the total number of samples, the normalized difference of 3% obtained before was only an absolute difference of one sample, i.e. the 27-parameter model correctly classified one more sample than the 7-parameter model.

This difference in classification was not as significant as the difference in classifier complexity: to correctly classify one more sample required a classifier with 4 times the number of entries. Thus, the most promising candidate in terms of accuracy and complexity was the ANN-based classifier with 7 input parameters.

### 4.4 Classifiers with best parameters from the previous work

After evaluating all the best classifiers with the most important variables, the next step was to evaluate those ML models with the set of most significant variables obtained in the previous work of [7], in order to compare both sets of parameters with the same technology. The final parameters selected in that work were: receiving psychological support; contact with the abuser; thinking of going back with the aggressor; feeling guilty; having a protection order and perceiving that the decisions of the judicial procedure are not made jointly with her lawyer.

Results of evaluation metrics obtained for each model for this set of parameters are shown in Table 12.

**Table 12:** Evaluation metrics for each classifier with set of previous work’s most important features.

	Accuracy	Sensitivity	Specificity	Precision	F1-score
**ANN**	87.07	0.6667	0.9298	0.7333	0.6984
**SVM_1_**	86.39	0.6970	0.9123	0.6970	0.6970
**SVM_2_**	86.39	0.6970	0.9123	0.6970	0.6970
**SVM_3_**	87.07	0.7273	0.9123	0.7059	0.7164
**RF**	86.39	0.7273	0.9035	0.6857	0.7059

**Fig 7:**
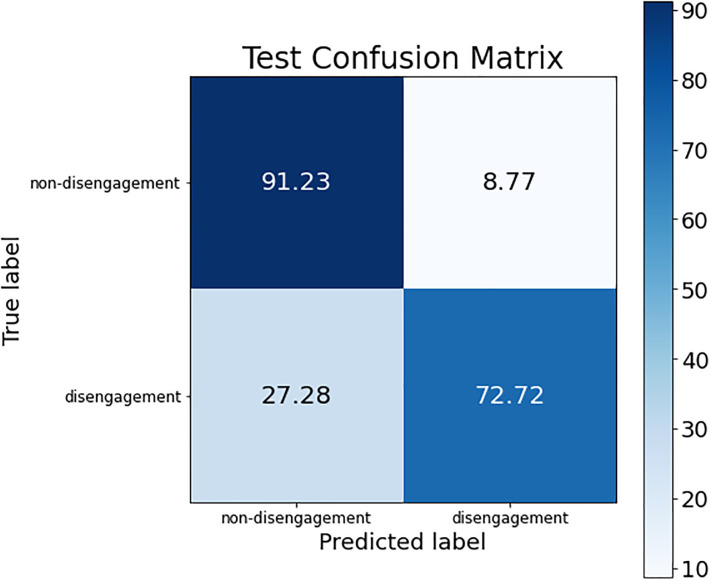
Confusion matrix for SVM_3_ model trained with the parameters used in the previous work.

As can be observed, the accuracy value obtained was lower than the one achieved before, and the sensitivity metric fell sharply to values of around 66–72%. For the best case of all of them (SVM_3_), the confusion matrix obtained is shown in Fig 7.

Compared with the previous simplified model, the non-disengagement class accuracy dropped only 1.75%, but the main problem remained in the disengagement class, whose accuracy value dropped more than 12%.

These results indicate that in the previous work, certain parameters were discarded from the dataset, and those parameters may improve the classification rate of the system. With this information, in the last subsection the difference in the parameter selection between the previous study and this new study was studied, and some combinations between those parameters were performed in order to determine which parameters (initially discarded in the previous work) could improve the classification.

### 4.5 Parameter combination

The last step of this comprehensive analysis of variables included the combination of the most important variables obtained for the different classifiers, together with those variables with a significant value obtained in the previous work.

After observing the similarities between the variables selected in the previous work and the most significant variables obtained in the “simplification of classifiers” subsection of this study, we obtained the results shown in Table 13.

**Table 13:** Variable comparison between previous study and the classifiers optimized for this work. Information presented in fraction mode, where the denominator represents the number of variables used for the row’s system, and the numerator represents the number of variables of the row’s system that matches the column’s system.

	**Prev. study**	**ANN**	**SVM**	**RF**
**Prev. study**	9/9	3/9	7/9	6/9
**ANN**	3/7	7/7	2/7	2/27
**SVM**	7/27	2/27	27/27	9/27
**RF**	6/30	2/30	9/30	30/30

The first column of Table 13 indicates the number of variables from each system that matches the variables selected in the previous work. For the best candidate selected in this work (ANN), there was a 43% coincidence in the variables selected.

The following study combined the variables used in the previous works with the new variables used in this study for each candidate. If the new systems were trained adding the variables one by one, millions of combinations would have to be performed; so the combinations were done by grouping the new variables in four subsets:

- Subset 1: containing those variables common in the three new systems (ANN, SVM and RF) that were not used in the previous work.
- Subset 2: containing those variables only used in ANN that were not used in the previous work.
- Subset 3: containing those variables only used in SVM that were not used in the previous work.
- Subset 4: containing those variables only used in RF that were not used in the previous work.

So, four new classifiers were trained combining the variables used in the previous work with the ones of each subset (named as “combined classifier” 1–4, or CC1–4 to simplify). The results obtained for testing those combinations in the ANN model are presented in Table 14.

**Table 14:** Combined classifiers’ results. Each row shows the results of the classifier trained with the previous work’s variables and the ones that contain the subset indicated in that row.

	Accuracy	Sensitivity	Specificity	Precision	F1-score
**CC1**	90.48	0.8182	0.9464	0.7714	0.7941
**CC2**	87.76	0.7272	0.9210	0.7272	0.7272
**CC3**	90.48	0.8182	0.9464	0.7714	0.7941
**CC4**	89.12	0.7575	0.9298	0.7353	0.7462

As can be observed in Table 14, the systems CC1 and CC3 obtained the same results. CC1 corresponded to the combination of the previous work’s variables and the common variables of the three new systems; while CC3 corresponded to the combination of the previous work’s variables and the variables only used for the SVM system.

The main difference between both of them was that CC1 included only 2 new variables, while CC3 included 21 new variables. Looking for the less complex system, the best candidate in this case was CC1.

The two new variables used in CC1 were: “plans to abandon” and “current questionnaire”. As there were only two, the next step was to analyze the inclusion of these variables one by one to ascertain whether both variables improved the previous work results or, on the contrary, only one of them did.

Table 15 illustrates the results of the classification, combining the variables used in the previous work with the common variable “plans to abandon”.

**Table 15:** Evaluation metrics for each classifier with set of previous work’s most important parameters combined with “plans to abandon”.

	Accuracy	Sensitivity	Specificity	Precision	F1-score
**ANN**	91.16	0.7878	0.9473	0.8125	0.8000
**SVM_1_**	89.11	0.6969	0.9473	0.7931	0.7419
**SVM_2_**	89.79	0.7273	0.9473	0.8000	0.7619
**SVM_3_**	89.11	0.7273	0.9386	0.7742	0.7500
**RF**	87.76	0.7273	0.9211	0.7273	0.7273

Table 16 illustrates the results of the classification, combining the variables used in the previous work with the common variable “current questionnaire”.

**Table 16:** Evaluation metrics for each classifier with set of previous work’s most important parameters combined with “current questionnaire”.

	Accuracy	Sensitivity	Specificity	Precision	F1-score
**ANN**	87.76	0.7273	0.9210	0.7273	0.7273
**SVM_1_**	86.39	0.6969	0.9123	0.6969	0.6969
**SVM_2_**	87.07	0.6969	0.9210	0.7187	0.7076
**SVM_3_**	87.07	0.7575	0.9035	0.6944	0.7246
**RF**	87.07	0.7273	0.9123	0.7059	0.7164

In the previous tables, three main findings are presented:

- Point 1: the inclusion of the variable “plans to abandon” improved the previous work’s classification from 87.07% to 91.16% (more than a 4% increase), obtaining the same global accuracy than the one presented in Table 9 for the best ANN parameters.
- Point 2: the inclusion of the variable “current questionnaire” did not lead to a significant improvement (only 0.69%).
- Point 3: the inclusion of both variables improved the previous work’s classification results, but obtained worse results than the ones obtained by including only the variable “plans to abandon”.

**Fig 8:**
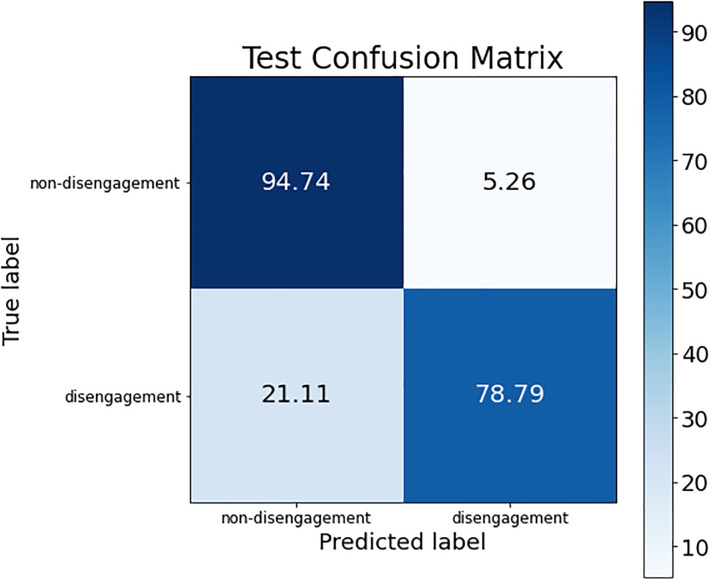
Confusion matrix for the final model obtained by the combination of the previous work’s variables and the new variable “plans to abandon”.

So, after analyzing the consequences of including the two new variables, the best solution was obtained by combining the previous work’s variables with the variable “plans to abandon”. The final results are detailed in its confusion matrix (see Fig 8).

## 5 Conclusions

In this work, different Machine Learning models were applied to predict disengagement from the legal proceedings by victims of IPVW, with the general purpose of obtaining a reliable support system so that the professionals can intervene before the withdrawal occurs.

For this purpose, three classifiers with different sets of the original dataset were tested, optimizing previously their hyperparameters and applying explainable AI techniques to obtain a subset of the most informative variables. This phase aimed to identify the model that best classified disengagement by the victim by using the minimum and most informative set of input parameters. Results have shown that the ANN-based classifier was the best candidate, identifying 7 variables as the most informative and obtaining an accuracy of 91.16%.

In addition, this work applied the ML classifiers to the set of variables used in the previous study (where a logistic regression model was used), to demonstrate that AI models obtained better accuracy results than the ones obtained in the previous study. The best result obtained in this case was for the SVM-based classifier with an accuracy of 87.07%, which was an improvement of more than 3%, compared to the accuracy of 83.6% obtained in the previous work.

In the last phase, by applying the different classifiers to the combination of the set of most informative variables with those obtained in the previous work, results showed that by adding one new variable (“plans to withdraw”) to the previous work’s BLR-based predictive models, the accuracy improved by 7.5%. This is a remarkable contribution to previous models for predicting withdrawals in IPVW cases. Knowing that the mere intention to withdraw is a good predictor of the final decision to desist from prosecution may be useful for professionals working with women victims, both in the legal arena and in psychosocial intervention, as it is an aspect that can be explored in women, after establishing a good victim-professional relationship based on confidence. This advanced warning for professionals about the risk of leaving the legal system and help them either prevent it when possible and advisable, or continue exploring possible aspects involved in this intention and on which it is possible to work to improve the situation of a woman who is involved within legal proceedings against her (ex)partner. From a theoretical view, considering the idea and actually going through with it to perform a behavior and the behavior itself -disengage from prosecution- may share psychological aspects that intervene in the victim’s cost-benefit assessment of continuing her participation in the legal proceedings [6, 8]. Yet, the fact that ML procedures have identified the intention to withdraw as one of the most informative variables on disengagement from prosecution has important implications for further research. Thus, new research questions arise, in the first place, over which variables could predict this intention to withdraw and therefore moderate their effect on the behavior of disengagement. Second, it would be interesting to explore, amongst the group of women who have declared the intention of withdrawing, the aspects and situations that differentiate between those women that do and those that do not eventually withdraw.

Overall, results obtained in this work showed that using ML-based classifiers for this problem and, thanks to the comparison work and the xAI studies, using one more variable to the previous work’s set, the accuracy results are improved substantially. Thus, in future studies, this new variable will be taken into account to reach more reliable predictions. However, it is worth noting that the dataset used for the detection of disengagement from the judicial process consists of 731 victims, which is a low number of samples for the model to make consistent and reliable predictions with new data. Therefore, future works should consider on extending the original dataset and make predictions by adding the new variable to the input parameters. Despite this limitation, the use of ML and the addition of new variables to the original works provides an additional benefit regarding the conceptualization of IPVW victims. These previous works defended the principle of parsimony which, despite its usefulness, may entail the risk reducing victims that withdraw from prosecution to specific features and the wrong idea of an existing IPVW victim profile. The different ML models implemented here have enriched the characteristics of the phenomenon studied and eased the understanding of the complexity of IPVW and victims’ decision-making processes.

Finally, future applications could include ML models being used to obtaining systems that allow more accurate detection of whether the victim will disengage from prosecution. Thus, through these support systems for professionals, it would be possible to detect some of the factors contributing to women’s higher likelihood to withdraw and therefore refer them to professional services to either prevent victims from disengaging or prepare them to face future legal proceedings. This could be done by developing a mobile or web application in which the predictor model is integrated. After including the answers to the initial forms answered by the victims before starting the judicial process, the professionals could obtain a prediction about the possible withdrawal and recommendations for action. In any case, and in line with [18], any professional tool based on artificial intelligence that is implemented must necessarily be complemented with sensitive professional assistance based on specialized training in the dynamics of IPVW and female victims’ decision-making processes.

## Clarifications

The interchangeable use of the terms “victim(s)” and “woman (women)” throughout the text is intentional because this study focuses on women who suffer violence by their male (ex)partners from a gender-based violence perspective. The participants in the study are all women, but this does not imply denying the existence of other forms of violence in which men may be the victims [6].

## Supporting information

S1 Appendix Variables in the dataset before the data pre-processing. (PDF)

## References

[1] Government Delegation for Gender Violence. Macrosurvey of violence against women 2019. (Ministerio de Igualdad, 2019), https://violenciagenero.igualdad.gob.es/violenciaEnCifras/macroencuesta2015/Macroencuesta2019/home.htm

[2] General Council of the Judiciary. Annual report on gender based violence: Year 2021. (Ministerio de Igualdad,2021), https://www.poderjudicial.es/stfls/ESTADISTICA/DOCUMENTOSCGPJ/Violencia%20sobre%20la%20mujer%20%20Informe%20Anual%20de%202021.pdf

[3] Dawson M. & Dinovitzer R. Victim cooperation and the prosecution of domestic violence in a specialized court. Justice Quarterly. 18, pp. 593–622 (2001) doi: 10.1080/07418820100095031

[4] Gauthier S. The Perceptions of Judicial and Psychosocial Interveners of the Consequences of Dropped Charges in Domestic Violence Cases. Violence Against Women. 16, pp. 1375–1395 (2010) doi: 10.1177/1077801210389163 21164215

[5] Buzawa E., Buzawa C. & Stark E. Responding to Domestic Violence: The Integration of Criminal Justice and Human Services. (SAGE Publications, 2017)

[6] García-Jiménez M., Durfee A., Cala-Carrillo M. & Trigo M. Psychosocial separation and women’s disengagement from prosecutions against abusive intimate partners in Spain. *Journal Of Interpersonal Violence*. 37, pp. NP9953–NP9980 (2022) doi: 10.1177/0886260520984424 33375875

[7] García-Jiménez M., Cala-Carrillo M. & Trigo M. Predicting disengagement from judicial proceedings by female victims of intimate partner violence in Spain: a systematic replication with prospective data. Violence Against Women. 26, pp. 1493–1516 31662100 10.1177/1077801219882502

[8] Kingsnorth R. & Macintosh R. Domestic violence: Predictors of victim support for official action. Justice Quarterly. 21, pp. 301–328 (2004)

[9] Robinson A. & Cook D. Understanding victim retraction in cases of domestic violence: Specialist courts, government policy, and victim-centered justice. Contemporary Justice Review. 9, pp. 189–213 (2006) doi: 10.1080/10282580600785017

[10] Sleath E. & Smith L. Understanding the factors that predict victim retraction in police reported allegations of intimate partner violence. Psychology Of Violence. 7, pp. 140–149 (2017) doi: 10.1037/vio0000035

[11] Cala M., Trigo E. & Saavedra F. Women’s disengagement from legal proceedings for intimate partner violence: Sociodemographic and psychological variables. European Journal Of Psychology Applied To Legal Context. 8, pp. 35–42 (2016) doi: 10.1016/j.ejpal.2015.10.002

[12] García-Jiménez M., Cala M., Trigo M. & De La Mata M. Women’s disengagement from legal proceedings for intimate partner violence in Southern Spain: Variables related to legal proceedings. Crime & Delinquency. 65, pp. 1873–1895 (2019) doi: 10.1177/0011128718789857

[13] García-Jiménez M., Cala M., Trigo M. & Barberá E. Indicators of liberation from gender-based intimate partner violence in Spain related to when charges are dropped. Psicothema. 32, pp. 40–46 (2020) 31954414 10.7334/psicothema2019.188

[14] Bzdok D., Altman N. & Krzywinski M. Statistics versus machine learning. Nature Methods. 15, pp. 233–234 (2018) doi: 10.1038/nmeth.4642 30100822 PMC6082636

[15] Escobar-Linero E., Domínguez-Morales M. & Sevillano J. Worker’s physical fatigue classification using neural networks. *Expert Systems With Applications*. pp. 116784 (2022) doi: 10.1016/j.eswa.2022.116784

[16] Civit-Masot J., Bañuls-Beaterio A., Domínguez-Morales M., Rivas-Pérez M., Muñoz-Saavedra L., & R. Corral J. M. Non-small cell lung cancer diagnosis aid with histopathological images using Explainable Deep Learning techniques. *Computer Methods and Programs in Biomedicine*. 226, pp. 107108 (2022) doi: 10.1016/j.cmpb.2022.107108 36113183

[17] Berk R., Sorenson S. & Barnes G. Forecasting Domestic Violence: A Machine Learning Approach to Help Inform Arraignment Decisions. Journal Of Empirical Legal Studies. 13, pp. 94–115 (2016,3) doi: 10.1111/jels.12098

[18] Grogger J., Gupta S., Ivandic R. & Kirchmaier T. Comparing Conventional and Machine-Learning Approaches to Risk Assessment in Domestic Abuse Cases. Journal Of Empirical Legal Studies. 18, pp. 90–130 (2021,3) doi: 10.1111/jels.12276

[19] Vijayakumar R. & Cheung M. Replicability of Machine Learning Models in the Social Sciences: A Case Study in Variable Selection. Zeitschrift Fur Psychologie / Journal Of Psychology. 226, pp. 259–273 (2018) doi: 10.1027/2151-2604/a000344

[20] Hindman M. Building Better Models: Prediction, Replication, and Machine Learning in the Social Sciences on JSTOR. The Annals Of The American Academy Of Political And Social Science, 659. pp. 48–62 (2015) doi: 10.1177/0002716215570279

[21] Walczak S. Predicting Crime and Other Uses of Neural Networks in Police Decision Making. *Frontiers In Psychology*. 12, pp. 587943 (2021,10) doi: 10.3389/fpsyg.2021.587943 34690848 PMC8529125

[22] Guerrero A., Cárdenas J., Romero V. & Ayma V. Comparison of Classifiers Models for Prediction of Intimate Partner Violence. Advances In Intelligent Systems And Computing. 1289, pp. 469–488 (2021) doi: 10.1007/978-3-030-63089-8_30

[23] Silva, J., Aleman, E., Acuña, G., Bilbao, O., Hernandez-P, H., Castro, B., et al. Use of artificial neural networks in determining domestic violence predictors. *Advances In Swarm Intelligence. ICSI 2019. Lecture Notes In Computer Science*. 11656 LNCS pp. 132–141 (2019)

[24] Rosili N., Zakaria N., Hassan R., Kasim S., Rose F. & Sutikno T. A systematic literature review of machine learning methods in predicting court decisions. IAES International Journal Of Artificial Intelligence (IJ-AI). 10, pp. 1091–1102 (2021,12) doi: 10.11591/ijai.v10.i4.pp1091-1102

[25] Ras G., Xie N., Gerven M. & Doran D. Explainable Deep Learning: A Field Guide for the Uninitiated. Journal Of Artificial Intelligence Research. 73, pp. 329–397 (2022) doi: 10.1613/jair.1.13200

[26] Acuña E. & Rodriguez C. The Treatment of Missing Values and its Effect on Classifier Accuracy. Classification, Clustering, And Data Mining Applications. pp. 639–647 (2004)

[27] Kaiser J. Dealing with Missing Values in Data. *Journal Of Systems Integration (1804-2724)*. 5 (2014)

[28] Garavaglia S. & Sharma A. A smart guide to dummy variables: Four applications and a macro. *Proceedings Of The Northeast SAS Users Group Conference*. 43 (1998)

[29] Ho, T. Random decision forests. Proceedings Of 3rd International Conference On Document Analysis And Recognition. 1, pp. 278–282 (1995)

[30] Boser, B., Guyon, I. & Vapnik, V. A training algorithm for optimal margin classifiers. Proceedings Of The Fifth Annual Workshop On Computational Learning Theory. pp. 144–152 (1992)

[31] Schmidhuber J. Deep learning in neural networks: An overview. Neural Networks. 61 pp. 85–117 (2015) 25462637 10.1016/j.neunet.2014.09.003

[32] Ribeiro, M., Singh, S. & Guestrin, C. Anchors: High-precision model-agnostic explanations. Proceedings Of The AAAI Conference On Artificial Intelligence. 32 (1) pp. 1527–1535 (2018)

[33] Noble W. What is a support vector machine?. Nature Biotechnology. 24, pp. 1565–1567 (2006) doi: 10.1038/nbt1206-1565 17160063

[34] Suthaharan S. Support Vector Machine. Machine Learning Models And Algorithms For Big Data Classification: Thinking With Examples For Effective Learning. pp. 207–235 (2016) doi: 10.1007/978-1-4899-7641-3_9

[35] Breiman L. Random forests. Machine Learning. 45, pp. 5–32 (2001)

[36] Sokolova M. & Others. A systematic analysis of performance measures for classification tasks. Inf. Process. & Manag. 45, pp. 427–437 (2009) doi: 10.1016/j.ipm.2009.03.002

