# Supplementary material for: Using machine learning-based systems to help predict disengagement from the legal proceedings by women victims of intimate partner violence in Spain
Source: PLoS One. 2023 Jun 7;18(6):e0276032. doi: 10.1371/journal.pone.0276032 (PMC10246857; doi:10.1371/journal.pone.0276032)
Supplement: S1 Appendix — (PDF) [file pone.0276032.s001.pdf]

# A Variables in the dataset before the data pre-processing

| Sociodemographic variables | Psychological, emotional and motivational variables | Variables related to the legal proceeginds and professionals |
|---|---|---|
| Age (in years) | Number of days that the woman has been suffering from IPVW | Data case: retrospective data, prospective data |
| Country of origin | The decision of filing the complaint: a well-thought decision, because of a traumatic event, another person filed the complaint, a well-thought decision + a traumatic event | Type of questionnaire administered: finalized proceedings, current proceedings, last previous complaint, for women who withdrew, questionnaires administered by CMIM/shelters/victims help foundations |
| Place of residence: rural, urban | The woman regrets having filed the complaint: no, yes | Court where the case was prosecuted |
| Number of children | The reason for filing a complaint was experiencing (no, yes): psychological violence, physical violence, sexual violence, economic violence, and other violence | Date of the initiation of the legal proceedings |
| Age in years of the first child (idem to sixth child) | Woman's expectations that (no, yes): the abuser stops abusing her, the abuser receives a scarce, that the abuser is imprisoned, getting a divorce, being protected, and other expectation | Date of the finalization of the legal proceedings by any reason |
| The income per month (in euros) | Contact with the abuser: none, occasional, frequent | Days since the legal proceedings started |
| Educational level: illiterate, compulsory and basic studies, professional training, bachelor's degree, university education | Type of contact (no, yes): through telephone, when handing over the children to their father, in family reunions, and other situations | Withdrawal by using Art. 416: no, yes |
| Spanish language knowledge: 0 = minimum, 10 = maximum | The woman had thought of the idea of going back with the abuser after she reported him: no, yes | The case was closed: no, yes |
| Any person in her family unit had a disability: no, yes | Going back because (no, yes): she loved him, for their children, to maintain the economic level, and other reasons | Service where the data was collected: SAVA Granada, SAVA Seville, CMIM, Courts |
| Who has the disability? | The woman has thought of withdrawing from prosecution: no, yes / The woman is sure she will withdraw: no, yes | Phase at the legal proceedings for the current complaint: first 72 hours, rapid trial, investigation phase, oral trial, finalized proceedings |

| | | |
|---|---|---|
| Any person in her family unit had an addiction: no, yes | Reason to withdraw (no, yes): economic reasons, fear, feeling pressured, she is tired, she wants him to leave her to live in peace, she is going back with him, and other reasons | Rapid trial: no, yes |
| Who has the addiction? | Feelings of (0 = not at all, 10 = completely): guilt after filing the complaint, fear about what he could do to her, fear about what he could do to their children, pressure to abandon the prosecution | The woman filed a previous complaint: no, yes |
| | If yes, who exerted such pressure? | Did she drop charges for a previous complaint?: no, yes |
| | Woman's assessment of the experience (0 = worst experience, 10 = best experience): when filing the complaint, with the Units of Assessment (for medical and expert reporting), during her declaration at the oral trial, during the trial, regarding the judge, regarding the prosecutor, and regarding her lawyer | The phase of the legal proceeding when the woman drops charges for such previous complaint: first 72 hours after filing the complaint, rapid trial, investigation phase, oral trial, finalized proceedings |
| | How much she (0 = minimum, 10 = maximum): is tired of the legal proceedings, has got the expected solution to her problem, is worried about money and/or employment, feels her life is in danger, has received support from family and/or friends, and feels strong to face the future | Where the woman filed the complaint: civil guard, national police, local police, courts |
| | The woman received psychological support: none, yes, through social services, yes, through health services | Level of knowledge the woman has about the legal proceedings: none, some, much |
| | Type of support: group therapy, individual therapy, both | Level of knowledge of the legal proceedings: none, some, much |
| | Who gave her support: SAVA, other social services, public health services, private health services, victims help foundations | Who gave her such information?: social services, SAVA, police officer/s, legal operators, the Media (TV, radio...), health services, others |
| | Number of days the woman has been receiving psychological support | With whom did the woman usually go to court?: alone, with one family person or friend, with more than one family person or friends, police officer/s |
| | What would you have needed and have not had during the process? | Divorce process: no, yes |

| | | | |
|---|---|---|---|
| | | Acceptance of romantic love myths (0 = totally disagree; 10 = totally agree): Love can do everything and is for life, I should not take care of myself until my family/partner is taken care of, I need a partner to feel like a complete woman, I still love him and, if he promised me to change, I would go back to him, Women are better able to take care of the family than men, Jealousy is a sign of true love | Whom did she feel that make the decisions during the legal proceedings? she alone, her lawyer alone, she jointly with her lawyer, her lawyer jointly with the abuser's lawyer |
| | | | Type of lawyer: private, public |
| | | | Imprisonment of the abuser: no, yes |
| | | | When she knew that he could be imprisoned: before the complaint, after the complaint, at the oral trial, when complaining the following time |
| | | | How much did she worry about the possibility of him being imprisoned? 0 = minimum, 10 = maximum |
| | | | The woman applied for a Protection Order (PO): no, yes |
| | | | The PO was granted: no, yes |
| | | | Appeal against the non-granting of a protection order: no, yes |
| | | | Type of protection order granted |
| | | | The woman acted as a private accusation: no, yes |
| | | | How well protected did she feel during the legal proceedings? 0 = minimum, 10 = maximum |

*Note: Out of the 116 variables in the dataset, we did not disclose here those referring to codes or registries for each participant and those that resulted from open-ended questions in the questionnaire that were not coded into categories. The output variable (withdraw: no, yes) has been omitted, too.*
